# Voluntary exercise increases striatal dopamine release and improves motor performance in aging mice

**DOI:** 10.1038/s41531-025-01213-7

**Published:** 2025-12-09

**Authors:** Guendalina Bastioli, Maria Mancini, Jyoti C. Patel, Begoña Gamallo-Lana, Jennifer C. Arnold, Adam C. Mar, Margaret E. Rice

**Affiliations:** 1 Department of Neuroscience, New York University Grossman School of Medicine, 435 E 30th Street, New York, NY 10016, USA; 2 Department of Neurosurgery, New York University Grossman School of Medicine, 435 E 30th Street, New York, NY 10016 USA

**Keywords:** Neuroscience, Physiology

## Abstract

Aging is often accompanied by a decline in mobility across species, which can be improved by aerobic exercise, even in individuals with Parkinson’s disease. We showed previously that 30 days of voluntary wheel-running exercise in young male mice leads to enhanced release of the motor-system transmitter, dopamine (DA), in ex vivo corticostriatal slices. Here we tested whether voluntary exercise also increases DA release in aging (12 months old) mice of both sexes, and whether this is associated with improved motor performance. Mice were allowed unlimited access to a rotating (runners) or a locked (controls) wheel for 30 days. Motor behavior was then assessed, and electrically evoked DA release was quantified in slices from these animals using fast-scan cyclic voltammetry. Although daily running distance for females was nearly twice that of males, runners of both sexes showed comparable increases in evoked DA release in dorsolateral striatum and in nucleus accumbens core and shell compared to age- and sex-matched controls. Runners of both sexes showed an increase in locomotion velocity and improved motor coordination. Thus, voluntary exercise boosts striatal DA release and improves motor performance in aging mice, providing new insights into the benefits of exercise in aging humans.

## Introduction

Aging is often accompanied by a decline in mobility and cognition^1^. Mobility issues involve physiological changes that include decreased dopamine (DA) transmission that affects motor and sensorimotor pathways^2–4^. The involvement of DA is unsurprising given the vital roles this transmitter plays in movement, reward, and cognitive function^5,6^. A key brain center for motor regulation is the striatum, which receives dense DA input from midbrain DA neurons in the substantia nigra pars compacta (SNc) and ventral tegmental area (VTA)^7–9^. Conditions in which DA is depleted, as when the nigrostriatal pathway degenerates progressively in Parkinson’s disease (PD), lead to motor impairment including deficits in the initiation, speed, and fluidity of voluntary movement that are hallmarks of PD^10,11^. Aging alone can be associated with similar deficits, however, and studies of the aging human brain have shown degraded DA signaling that is correlated with slower movement and decreased movement amplitude^12,13^.

It is well-recognized that aerobic exercise can be beneficial for brain health, including in the aging population^14–17^. In humans and in animal models, physical activity has been shown to enhance adult hippocampal neurogenesis, synaptic plasticity, and neurotrophin levels that contribute to improved memory^17–19^. Physical exercise also provides neuroprotection for brain DA pathways in neurotoxin-based PD models in rodents and improves motor and/or cognitive function in these animal models^20–29^. Moreover, aerobic exercise has been shown to improve motor activity in human PD patients^30–33^. Complementing and extending this body of evidence, we recently reported that voluntary exercise leads to an increase in dynamic DA release throughout the striatum of young, healthy male mice and that this requires a neurotrophin, brain-derived neurotrophic factor (BDNF)^34^. These results suggest that the motor benefits of exercise seen in humans, including those with PD, may involve increased DA release. However, whether amplified DA release in rodents is associated with improved motor performance, and whether these benefits can be extended to the aging brain have not been investigated.

Here, we evaluated the influence of physical exercise on striatal DA release, as well as on mobility and motor coordination in aging (12 months old) male and female mice. Mice were allowed unlimited access to a freely rotating wheel (runners) or a locked wheel (controls) for 30 days. Electrically evoked DA release was quantified using fast-scan cyclic voltammetry (FSCV), in the dorsolateral striatum (dlStr) and nucleus accumbens (NAc) core and shell in ex vivo corticostriatal slices from these animals. Given that striatal DA release can be driven by acetylcholine (ACh) released from cholinergic interneurons (ChIs) and acting at nicotinic ACh receptors (nAChRs) on DA axons^35–41^, we also assessed possible contributions from changes in ACh-dependent regulation of DA release. Behavioral testing was conducted in the same mice used for DA release studies, and included measures of locomotor activity in the open field, time to descend a vertical pole, and grip strength. Daily running activity was monitored for the exercise cohorts, along with weekly assessment of body weight and food consumption for all mice. Consistent with data from our earlier studies in young male mice, voluntary exercise led to increased DA release in striatal slices from aging mice of both sexes. Moreover, this was associated with an increase in the velocity of locomotor activity and improved motor coordination.

## Results

### Running activity in female and male mice

**Fig. 1:**
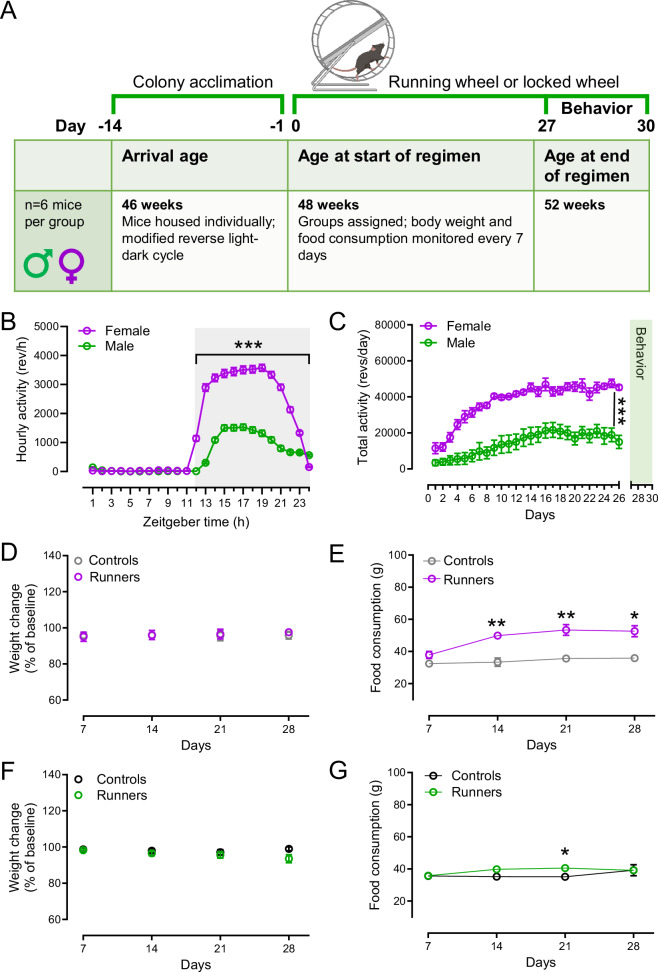
Voluntary wheel running. **A** Timeline for wheel-running protocol. For each study, 12 singly housed mice of a given sex were acclimated to a modified light-dark cycle for two weeks, then randomly assigned to cages with a freely rotating wheel (runners, *n* = 6) or a locked wheel (controls, *n* = 6). Behavioral testing was conducted on days 28 and 29, then on day 30, mice were removed for brain slice studies of evoked DA release using FSCV. Mouse image created in https://BioRender.com. **B** Daily running patterns for each sex, with greatest activity in the dark phase for both sexes (shaded box). Females showed greater activity in revolutions per hour (revs/h) than males (***p < 0.001; 2-way ANOVA, Sidák’s *post hoc* test, *n* = 6 per sex). **C** Average total daily running patterns also showed that females showed greater daily activity (revs/day) than males during the first 27 days that were uninterrupted by behavioral testing (***p < 0.001; 2-way ANOVA, Sidák’s *post hoc* test, *n* = 6 runners per sex). **D, F** Change in body weight monitored weekly, with the first day of housing with a wheel taken as baseline (100%). No significant changes in weight were seen in or between any group (female or male runners and controls (*n* = 6 per group; 2-way ANOVA, Dunnett’s and Sidák’s *post hoc* test). **E, G** Average food consumption for female and male cohorts assessed weekly (*p < 0.05, **p < 0.001 female runners vs. female controls, *p < 0.05 male runners vs. male controls, *n* = 6 mice per group; 2-way ANOVA, Sidák’s *post hoc* test).

Voluntary wheel-running activity over 30 days was monitored for singly housed male and female C57Bl6/J mice that were 12 months old at the end of the running period. Data from runners were compared with those from age and sex-matched controls that were singly housed with a locked wheel (Fig. 1A). Mice allowed access to a freely rotating wheel ran primarily during the dark phase, which began at 12:00 Zeitgeber time (Fig. 1B). In these studies, average running activity during the daily dark cycle for females was significantly greater than for males (F(1,369) = 293.5, *p* < 0.0001, 2-way ANOVA with repeated measures, *p* < 0.0001 for 12 to 24 h, Sidák’s *post hoc* test; females *vs*. males, *n* = 6 mice per sex) (Fig. 1B). Higher daily running activity in females *vs*. males was seen from the third day of wheel access and persisted through the rest of the running period; illustrated data show activity through day 26, after which the running period was interrupted intermittently for behavioral testing (F(1,10) = 46.75, *p* < 0.0001, 2-way ANOVA with repeated measures; *p* < 0.05 to *p* < 0.01 for days 4-26, Sidák’s *post hoc* test; females *vs*. males, *n* = 6 mice per sex) (Fig. 1C, Supplementary Table 1). Body weight and food consumption were assessed once a week. Initial body weights were ~29 g for females and ~35 g for males. When compared to these initial weights, there was no change in weight for any group. Moreover, there were no differences between runners *vs*. controls for females or males (female: (F(1,10) = 0.0251, *p* = 0.8772; male: (F(1,10) = 2.532, *p* = 0.1426 2-way ANOVA with mixed effects model, *p* > 0.05 at 7, 14, 21 and 28 days Sidák’s *post hoc* test, *n* = 6 mice per group) (Fig. 1D, F, Supplementary Table 1). However, maintenance of weight in female runners was associated with increased food consumption after the second week (F(1,10) = 38.89, *p* < 0.0001, 2-way ANOVA, mixed effects model, *p* < 0.01 at 14 days, *p* < 0.01 at 21 days and *p* < 0.05 at 28 days, Sidák’s *post hoc* test; runners *vs*. controls, *n* = 6 mice per group) (Fig. 1E). Male runners showed an increase in food consumption only in their third week (F(1,10) = 2.179, *p* = 0.1707, 2-way ANOVA, mixed effects, *p* < 0.05 at 21 days, Sidák’s *post hoc* test; runners *vs*. controls, *n* = 6 mice per group) (Fig. 1G, Supplementary Table 1).

### Voluntary exercise increases evoked DA release in aging female and male mice

**Fig. 2:**
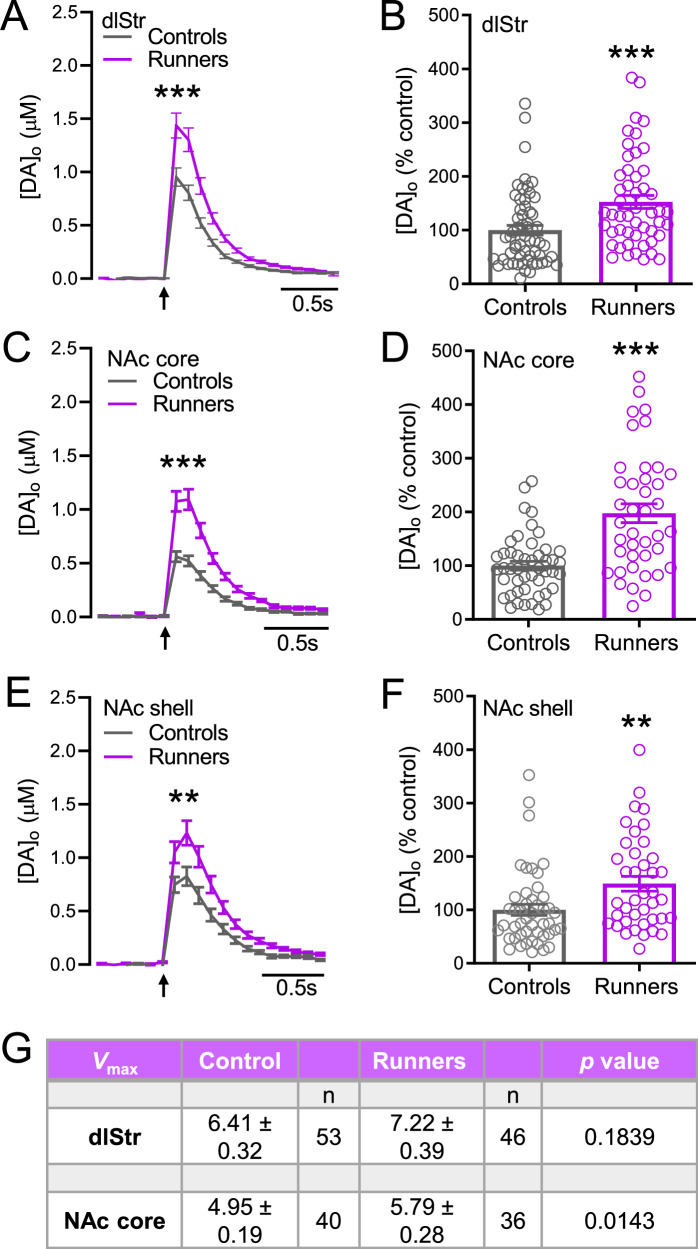
Increased evoked [DA]_o_ in striatal slices from female mice, after 30 d of voluntary wheel running. **A, C, E** Averaged evoked increases in [DA]_o_ with SEM in dStr and NAc core (single-pulse stimulation) and NAc shell (5 pulses, 100 Hz) in ex vivo slices from female runners and controls. Arrows indicate time of stimulation. **B, D, F** Data summary for evoked [DA]_o_ in each region for runners *vs*. controls, normalized to mean peak [DA]_o_ for each region in controls. **A**–**F** *n* = 40–59 sites/region, 2 slices/mouse, 6 mice/group; **p < 0.01, ***p < 0.001; unpaired *U-*tests. **G** *V*_max_ values derived for evoked striatal DA release using a fixed *K*_m_ of 0.9 µM in control female mice and in female runners. Data are means ± SEM and were analyzed using a *U*-test for dlStr and an unpaired *t*-test with Welch’s correction for NAc core (*p < 0.05; *vs*. control); data with a goodness of fit *R*^2^ < 0.90 were excluded.

**Fig. 3:**
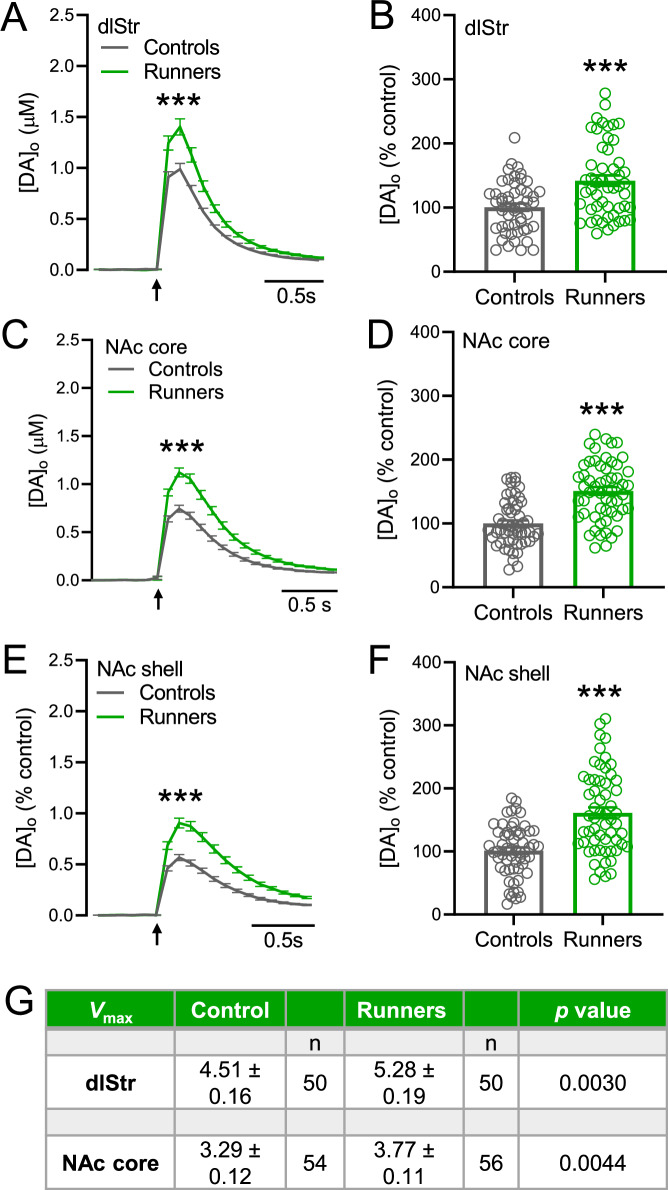
Increased evoked [DA]_o_ in striatal slices from male mice after 30 d of voluntary wheel running. **A, C, E** Averaged evoked increases in [DA]_o_ with SEM in dStr, NAc core (single-pulse stimulation) and NAc shell (5 pulse, 100 Hz) in ex vivo slices from male runners and controls. Arrows indicate time of stimulation. **B, D, F** Data summary for evoked [DA]_o_ in each region for runners *vs*. controls, normalized to mean peak [DA]_o_ for each region in controls. **A**–**F** *n* = 50–59 sites/region, 2 slices/mouse, 6 mice/group; ***p < 0.001; unpaired *t-*tests. **G** *V*_max_ values derived for evoked striatal DA release using a fixed *K*_m_ of 0.9 µM in control male mice and in male runners. Data are means ± SEM and were analyzed using an unpaired *t*-test for each region (**p < 0.01 *vs*. control); data with a goodness of fit *R*^2^ < 0.90 were excluded.

The influence of exercise on dynamic DA release in the striatum was assessed using multiple-site sampling of locally evoked extracellular DA concentration ([DA]_o_) in dlStr, NAc core, and NAc shell in corticostriatal slices from runners and controls. We found that voluntary exercise led to a significant increase in evoked [DA]_o_ in each striatal subregion in aging runners of both sexes *vs*. age and sex-matched controls (Figs. 2 and 3). Notably, these increases were comparable to the exercise-induced increases in striatal DA release reported previously for young (10-week-old) male mice^34^. In aging females, mean evoked [DA]_o_ in the dlStr of controls was 0.96 ± 0.08 µM and 1.46 ± 0.11 µM in runners (Fig. 2A, B); in NAc core, control evoked [DA]_o_ was 0.58 ± 0.04 µM and 1.15 ± 0.10 µM in runners (Fig. 2C, D); and in NAc shell, control evoked [DA]_o_ was 0.83 ± 0.08 µM and in runners was 1.24 ± 0.11 (Fig. 2E, F) (dlStr, *p* < 0.001 runners *vs*. controls, *n* = 50−59 sites from 6 mice per group, unpaired *U-*test; NAc core, *p* < 0.0001, *n* = 40−47 sites from 6 mice per group, unpaired *U-*test; NAc shell, *p* < 0.01, *n* = 40−47 sites from 6 mice per group, unpaired *U-*test). Although less precise, comparing evoked [DA]_o_ averaged across slices rather than recording sites also showed significant exercise-enhanced DA release in all striatal subregions of female runners *vs*. controls (*n* = 12 slices per group; Supplementary Fig. 1A-C). Voluntary exercise in female mice was not associated with a change in the maximal rate (*V*_max_) for DA uptake in dlStr, but did lead to a significant increase in *V*_max_ in the NAc core with respect to controls (Fig. 2G) (dlStr, *p* = 0.1839, runners *vs*. controls, *n* = 46−53, unpaired *U*-test; NAc core *p* < 0.05, *n* = 36−40, unpaired *t-*test with Welch’s correction).

Despite running significantly less each day than females, aging male runners also showed a significant increase in evoked [DA]_o_ *vs*. matched controls (Fig. 3). In aging males, mean evoked [DA]_o_ in dlStr was 0.99 ± 0.05 µM in controls and 1.40 ± 0.07 µM in runners (Fig. 3A, B); in NAc core, evoked [DA]_o_ was 0.75 ± 0.03 µM in controls and 1.12 ± 0.04 µM in runners (Fig. 3C, D); and in NAc shell, evoked [DA]_o_ was 0.57 ± 0.03 µM in controls and 0.91 ± 0.05 in runners (Fig. 3E, F) (dlStr, *p* < 0.001 runners *vs*. controls, *n* = 51–50 sites from 6 mice per group, unpaired *t-*test; NAc core, *p* < 0.0001, *n* = 56–55 sites from 6 mice per group, unpaired *t-*test; NAc shell, *p* < 0.0001, *n* = 59–58 sites from 6 mice per group, unpaired *t-*test). As seen in females, a significant increase in evoked [DA]_o_ was seen for each striatal subregion from male runners *vs*. controls when averaged across slices rather than sites (*n* = 12 slices per group; Supplementary Fig. 1A-C), as seen in females. Analysis of *V*_max_ in males showed an increase in *V*_max_ for DA uptake in runners compared to controls in both dlStr and NAc core (Fig. 3G) (dlStr, *p* < 0.01, runners *vs*. controls *n* = 50 for both; NAc core *p* < 0.01 *n* = 54–56, unpaired *t-*test). Given that enhanced DA uptake acts to lower [DA]_o_, these findings indicate that the net effect of voluntary exercise on [DA]_o_ in both males and females is to enhance DA release.

### Absence of ACh involvement in exercise-enhanced striatal DA release

**Fig. 4:**
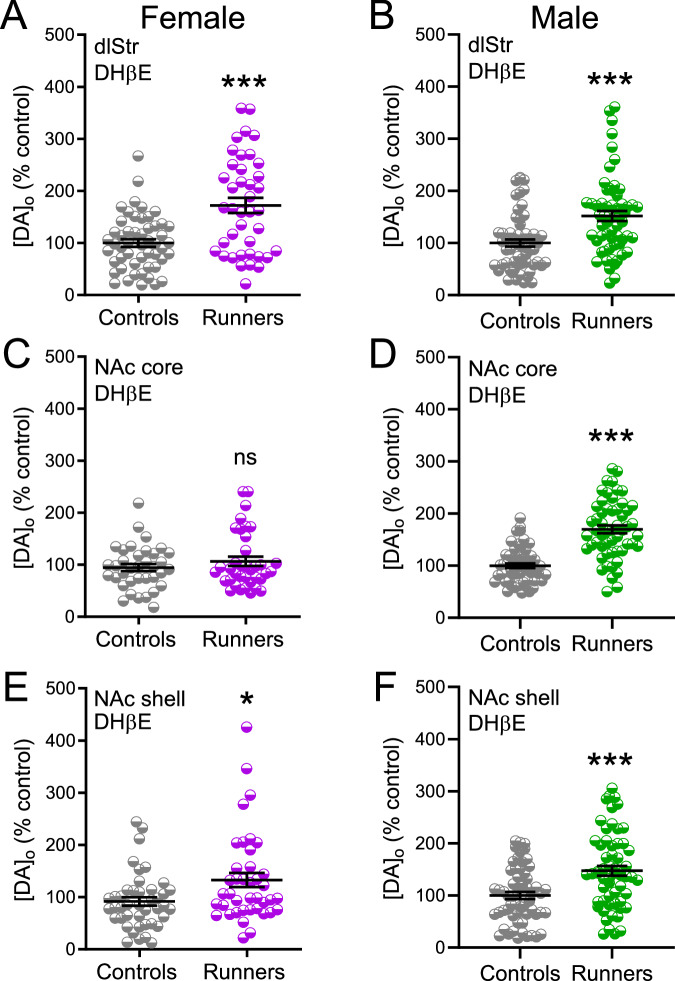
Evoked [DA]_o_ in striatal slices from female and male runners and controls in the presence of a nAChR antagonist. Data summary for dlStr, NAc core, and NAc shell from female (**A, C, E**; fuchsia) and male (**B, D, F**; green) runners and controls. Data are normalized to mean peak evoked [DA]_o_ in controls for each region in DHβE (1 µM) (*n* = 38–60 sites/region, 2 slices/mouse, 6 mice/group; ns not significant, *p < 0.05, ***p < 0.001; unpaired *U*- or *t-*tests).

Having established that exercise boosts striatal DA release in aging mice of both sexes, we next asked whether the effect involved altered DA release regulation by ACh and nAChR activation. It is well-recognized that activation of nAChRs can trigger striatal DA release^35–41^, and that this can contribute to DA release enhancement by external regulators, including insulin and leptin^42–44^. We investigated a role for ACh in exercise-enhanced DA release using a nAChR antagonist, dihydro-β-erythroidine (DHβE; 1 µM)^34,35^, to remove the contribution of nAChR activation to evoked DA release. In contrast to the DA-boosting effects of insulin and leptin, the observed exercise-induced enhancement of DA release persisted when nAChRs were antagonized in all but one striatal subregion in one sex, indicating independence from nAChR activation and implying a direct effect of exercise on DA axons (Fig. 4). The exception was in the NAc core of female runners, as discussed further below. In both sexes, whether runners or controls, antagonism of nAChRs by DHβE decreased the amplitude of evoked [DA]_o_ in regions in which single-pulse stimulation was used to evoke DA release, as previously^34,35,38^. In the dlStr of females and males, evoked [DA]_o_ remained significantly higher in runners than controls (Fig. 4A, B) (*p* < 0.001 runners *vs*. controls, *n* = 52–42 sites from 6 female mice per group; *n* = 60 sites per group from 6 males per group; unpaired *U-*tests). In NAc core of females, the difference between runners and controls was absent in the presence of DHβE (*p* = 0.973 runners *vs*. controls, *n* = 39–38 sites from 6 female mice per group, unpaired *U-*test) (Fig. 4C), whereas higher evoked [DA]_o_ persisted in the NAc core of male runners (*p* < 0.001 runners *vs*. controls, *n* = 55–56 sites per group from 6 male mice per group, unpaired *t-*test) (Fig. 4D). Elevated evoked [DA]_o_ also persisted in the NAc shell of runners of both sexes (Fig. 4E, F) (females, *p* < 0.05 runners *vs*. controls, *n* = 40–45 sites from 6 mice per group, unpaired *U-*test; males, *p* < 0.001 *vs*. controls, *n* = 60–60 sites from 6 mice per group, unpaired *U-*test).

### Wheel-running exercise improves motor function in aging mice

**Fig. 5:**
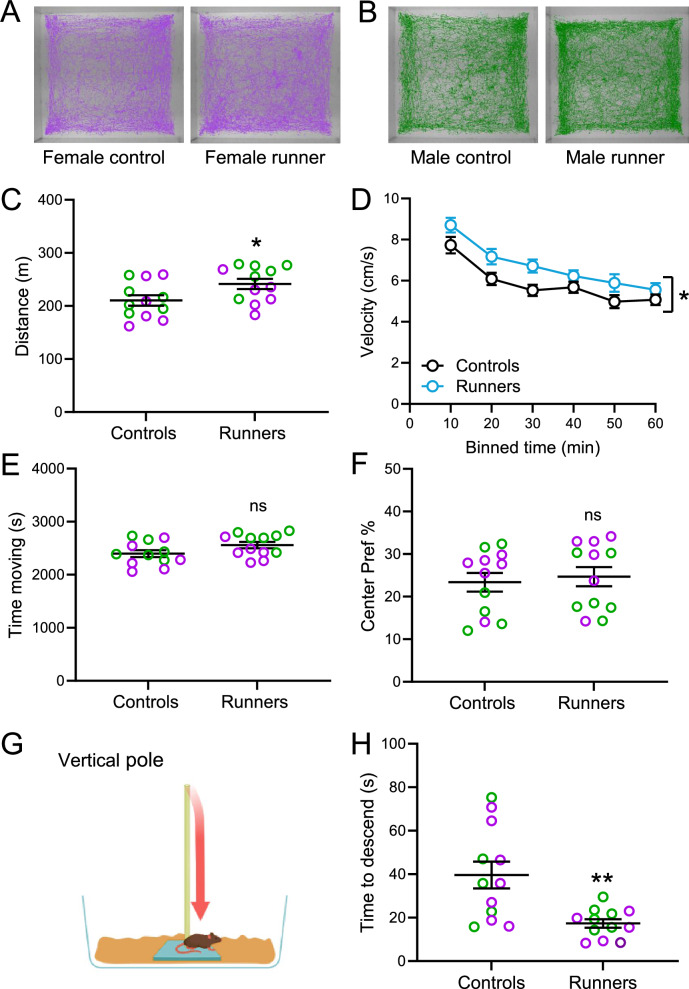
Increased motor speed and improved motor coordination in runners *vs*. controls. **A, B** Example of locomotor behavior in the open field for a female control and female runner and a male control and male runner recorded over 1 h. **C** Distance traveled for runners *vs*. controls (pooled data from both sexes, females in fuchsia, males in green) (*p < 0.05 runners *vs*. controls, *n* = 12 mice/group; unpaired *t-*tests). **D** Movement velocity (pooled data from both sexes) averaged in 10-min bins (runners *vs*. controls, *n* = 12 mice/group; 2-way ANOVA RM, Sidák’s *post hoc* test). **E** Time spent moving over 60 min for runners and controls (pooled data from both sexes; female in fuchsia, male in green) (ns not significant; *n* = 12 mice/group; unpaired *t-*tests). **F** Time spent in the center of the open field over 60 min of observation for runners and controls (pooled data from both sexes; female in fuchsia, male in green) (*n* = 12 mice/group; unpaired *U-*tests). **G** Pole test diagram. **H** Time to descend pole for runners and controls (pooled data from both sexes; female in fuchsia, male in green) (**p < 0.01, *n* = 12 mice/group; unpaired *t-*tests).

Our previous studies showed that exercise boosts DA release in young male mice^34^. Our current results show that voluntary exercise leads to enhanced striatal DA release in aging mice of both sexes, as well. We therefore tested the hypothesis that amplified DA release might contribute to improved motor performance in aging mice. We tested motor behavior in runners and controls during the last three days of the running period. We first examined locomotor behavior in an open field arena (Fig. 5A, B). The overall distance traveled in the open field across a 60-min test period (females and males combined), was significantly greater for runners than controls (*p* < 0.05, 12 mice per group, unpaired *t-*test) (Fig. 5C), showing greater velocity of movement in runners over 60 min, with a significant main effect of exercise, although analysis of individual 10-min bins did not reveal a significant exercise group x time bin interaction (F(1,22) = 5.191, *p* = 0.0328 for total exercise; *p* > 0.05 for 10-min bins, *n* = 12 mice per group, 2-way ANOVA, Sidák’s *post hoc* test) (Fig. 5D, Supplementary Table 2). The overall time spent moving did not differ between runners and controls (*p* = 0.084, *n* = 12 mice per group, unpaired *t-*test) (Fig. 5E). Monitoring mice in the open field also allowed evaluation of the time each mouse spent in the center *vs*. perimeter of the arena as a measure of anxiety-like behavior. We found no difference in the time spent in the center of the arena between runners and controls, consistent with a limited anxiety phenotype in healthy aging mice, whether engaged in aerobic exercise or not (*p* = 0.347; 12 mice per group, unpaired *U*-test (Fig. 5F).

We next assessed motor performance in a pole test. Evaluation of time to descend a vertical pole (Fig. 5G) revealed a significantly shorter time to descend the pole in runners compared to controls, indicating a marked improvement in motor coordination in runners (*p* < 0.01; 12 mice per group, unpaired *t*-test) (Fig. 5H). The adhesive tape removal test was used to assess dexterity and fine motor coordination. In contrast to the motor improvements seen in the open field and pole tests, this test showed that although control females were slightly faster to approach the first tape for removal than their male counterparts (F(1,20) = 13.420, *p* = 0.0015 for sex *p* < 0.05 for female controls *vs*. male controls, *n* = 6 mice per group, 2-way ANOVA, Sidák’s *post hoc* test), there was little difference between runners and controls, with no effect of exercise on time to first approach in either females or males (F(1,20) = 0.4855, *p* = 0.4940 for exercise; *p* = 0.9985 for female controls vs. runners, and *p* = 0.9230 for male controls vs. runners, *n* = 6 mice per group, 2-way ANOVA, Sidák’s *post hoc* test) (Supplementary Fig. 2A, Supplementary Table 2) and no significant change in the time to remove the first tape (Supplementary Fig. 2B) or the second tape (Supplementary Fig. 2C) in males and females combined (*p* = 0.109; *p* = 0.105; 12 mice per group, unpaired *t*-test). We also found no difference in forelimb or combined forelimb and hindlimb grip strength between runners and controls (Supplementary Fig. 2D,E) (*p* = 0.787; *p* = 0.488*;* 12 mice per group, unpaired *t*-test). Overall, these results show that voluntary exercise can counteract the effects of aging to improve motor function without corresponding improvement in fine motor skills or strength.

## Discussion

Aging adults experience a decline in mobility involving physiological changes in the brain that include decreased DA transmission which affects motor and sensorimotor pathways^2,3^. Exercise has long been recognized to benefit human health^45^. Beyond well-recognized benefits for the cardiovascular system^46,47^, exercise can improve human brain health, with benefits that include improved cognition and memory^16,48,49^, improved motor function in healthy aging adults and improved motor function in those with PD^30–32,50^. However, the cellular and circuit mechanisms involved in the beneficial effects of exercise are not yet well understood. Our previous studies in young male mice showed that 30 days of voluntary wheel-running exercise boosts evoked striatal DA release^34^. Notably, in that study, we found that exercise-enhanced DA release persists in the dlStr and NAc core after 7 days of rest (Bastioli et al. 2022). This indicates that the increase in evoked [DA]_o_ is not from the act of running per se but rather is an enduring consequence of exercise. Here we report that aerobic exercise can also enhance striatal DA release in aging males and in females. Moreover, we found that this is associated with improved motor performance, including increased locomotor speed and improved coordination.

Voluntary exercise increased evoked DA release in all striatal subregions in both sexes. Interestingly, female mice ran more than twice the daily distance of age-matched males, consistent with previous studies showing greater activity in females^51,52^. Nevertheless, there was enhanced evoked DA release in both sexes, with comparable increases seen in males and females (Figs. 2 and 3), and comparable behavioral improvements, as well. Given the far greater activity of females *vs*. males, this implies that a threshold level of activity was met in both sexes to produce benefits.

Previous studies have established that female mice and rats run faster and farther than males, particularly at younger ages^52–55^. However, it has been suggested sex differences in running behavior decline with aging. For example, Bartling and colleagues found that although female mice exhibit more running activity than males when young, this difference diminishes by 9 months with continued access to the running wheel^56^. Our data, however, show that sex differences in running activity are clearly evident in older mice when running wheels are available for 30 days rather than throughout the aging process. It is relevant to note that there are also strain differences in the propensity to run^53^ that might contribute: Bartling et al. (2016) examined C57Bl/6N and the present study examined C57Bl/6J. Higher wheel-running activity in females than males remains incompletely explained. Implicated factors include circulating sex hormones and estrogen receptors^54,55^, although contributions from these would be expected to diminish by 12 months of age in C57Bl/6J females^57^. Other previously reported sex differences include respiratory capacity, food intake and energy metabolism^58,59^, each of which could impact running activity^60^. In our study, female runners showed increased food consumption over the 30-day running period without weight gain, whereas no other group, including male runners, showed such marked changes in either consumption or weight. Although this observation in females might suggest a change in physiological processes governing food intake and energy metabolism with exercise, it may simply reflect normal, homeostatic balancing of energy intake and expenditure.

It is well-established that striatal DA release can be driven by ACh acting at nAChRs on DA axons^35–41^. To test a role for cholinergic involvement in the influence of exercise on evoked [DA]_o_, we compared absolute evoked [DA]_o_ in striatal slices from runners and controls in the presence of the nAChR antagonist, DHβE. Consistent with our results from young male mice^34^, we found that exercise-induced enhancement of evoked [DA]_o_ was independent of cholinergic involvement with persistence of higher DA release in runners vs. controls even with nAChR antagonism. This demonstrates a cell-autonomous effect of voluntary exercise on evoked increases in [DA]_o_ in both sexes. Moreover, we show that this reflects enhanced axonal DA release rather than decreased DAT-mediated uptake. Indeed, our *V*_max_ analysis revealed slight but significant *increases* in striatal DA uptake that would slightly temper the dominance of exercise-enhanced DA release to evoked [DA]_o_.

The mechanism by which exercise enhances DA release is not yet established, although we do know from our previous work in young males that this requires BDNF and is absent in mice that have heterozygous BDNF deletion (BDNF^+/-^)^34^. The main target of BDNF in the brain is the tropomyosin receptor kinase B (TrkB) receptor^61–63^. We find that TrkB agonist application in slices increases evoked [DA]_o_ throughout the striatum, implying that BDNF elevation is not only necessary, but also sufficient for DA release amplification^34^. These data are in line with earlier work showing that exogenous BDNF enhances [^3^H]DA overflow from rat striatal tissue^64,65^ and increases evoked [DA]_o_ in slices from BDNF^+/-^ mice^66^. Notably, BDNF levels in SNc and striatum fall in PD^67–69^, and neuroprotective effects of exercise after MPTP^70^^,^ are lost in BDNF^+/-^ mice^70,71^.

Our investigations of the effect of exercise on DA release were initially motivated by increasing evidence of motor improvement in PD patients who exercise. A variety of exercise programs have been shown to increase the health-related quality of life (HRQOL) status of individuals with PD^32^. Improvements have been reported in motor and also non-motor symptoms of PD^25,32,72–83^. In rodent toxin models of PD, exercise has been shown not only to improve motor outcome, but also to slow DA neuron loss^20–29,70,84,85^. Consistent with evidence for neuroprotection is the role identified for neurotrophic factors, including BDNF, as key players in the benefits of exercise^14,34,71,86–90^.

The studies in aging mice reported here represent a key step in understanding a role for exercise-enhanced DA release in motor improvements in PD. Aging is frequently associated with a decline in mobility, as well as cognition^2–4^. To assess the impact of exercise on mobility and other aspects of movement, we conducted several distinct motor-related behavioral tests on runners and controls. Consistent with the observed increase in DA release, we found that 30 days of exercise increased the average velocity of locomotor activity in the open field. Runner mice also descended a vertical pole roughly twice as fast as controls. Thus, exercise improved mobility and coordination in runners compared to control mice. In contrast, dexterity, fine motor coordination and strength were unaffected in the same cohorts of mice. The lack of change in grip strength is informative, as it suggests that the observed increase in locomotor speed is not simply a consequence of increased muscle strength from the month of wheel-running exercise. This pattern of motor benefits is strikingly similar to that seen in PD patients who have exercise therapy *vs*. no intervention or placebo, who show improvement in gait outcomes, including speed, and also in balance^31,91^.

The critical role of DA in restoring motor function is clearly demonstrated in the efficacy of L-DOPA, the primary treatment for PD, which is a DA precursor molecule that helps boost DA levels and function in humans and in rodent models of PD^41,92^. However, L-DOPA does not slow disease progression and eventually leads to debilitating DOPA-induced dyskinesia^92–95^. Consequently, the development of new directions for PD treatment and age-dependent motor decline in general is critical. Previous studies have found that in aging mice, long-intensity or mild-intensity physical exercise programs can improve cognitive functions and synaptic plasticity^96–98^, but short-term exercise programs using a treadmill do not^99^. These findings point to the importance of developing appropriate exercise programs to obtain improvements in motor and cognitive functions in the human population, whether for healthy aging or as adjunct therapy for PD or other disorders^48,78,83,100^. Current evidence implicates loss of DA as a contributing factor to aberrant plasticity underlying motor dysfunction in PD^29,50,101,102^ with reversal of this following DA replacement^103^. Given these results, as well as the established role of DA in striatal circuitry and plasticity^104,105^, our findings implicate exercise-enhanced endogenous DA release as a contributing factor in motor improvements seen with exercise, not only in mice, but also in aging humans.

## Methods

### Animal handling

Studies were conducted using male and female C57Bl/6J mice, 46-52 weeks of age, obtained from The Jackson Laboratory (JAX stock No. 000664). Animal procedures were in accordance with the National Institutes of Health guidelines and approved by the New York University Grossman School of Medicine Animal Care and Use Committee.

### Housing and voluntary wheel-running exercise

For the wheel-running paradigm, mice were 46 weeks old upon arrival, and 52 weeks old at the end of the study. Cohorts of 12 male or 12 female mice were examined. Immediately after arrival, mice were housed individually in a modified 12 h reverse light/dark cycle, with lights off from 10:00 a.m. to 10:00 p.m. local time, with *ad libitum* access to food and water. After 14 d of acclimation to the light/dark cycle (Fig. 1A), mice were randomly assigned at staggered intervals to either runner (freely rotating wheel) or control (locked wheel) groups and given unlimited, voluntary access to the wheel and to food and water for 30 d, as in previous studies^34,87,106^ (Fig. 1A). All behavioral testing and striatal slice recording of evoked DA release was conducted in the early hours of the dark phase when mice are most active (*e.g*., Fig. 1B).

Custom vertical running wheels (11.5 cm diameter, 5.8 cm width), developed by the NYU Langone Rodent Behavior Core, were utilized within universal InnoVive home caging, as previously^34^. The wheels were wire mesh, mounted on a 3D-printed suspension with low-torque double-shielded ball bearings. Rotational events were detected using a 5 mm neodymium magnet attached to the wheel paired with a calibrated Hall effect sensor (A3144) mounted to the suspension. Data acquisition was managed using Arduinos programmed with custom code (by ACM) to ensure precise and high-speed measurement of rotation events, which allowed monitoring of running activity (indicated by number of wheel revolutions) across the light-dark cycle. Body weight and food consumption were assessed for each subject at the beginning of each study, then once weekly thereafter. Mice were housed continuously with wheels in their home cages, except when they were removed briefly for weekly weighing or for motor behavior testing on days 27- 30 (Fig. 1A).

### Dopamine recording using FSCV

Procedures for preparing ex vivo brain slices were as described previously^34,38,44,107^. Each animal was deeply anesthetized with isoflurane then the brain removed and placed into ice-cold HEPES-buffered artificial cerebrospinal fluid (aCSF)^34,35^ containing the following (in mM): 120 NaCl, 20 NaHCO_3_, 10 glucose, 6.7 HEPES acid, 5 KCl, 3.3 HEPES sodium salt, 2 CaCl_2_, and 2 MgSO_4_, equilibrated with 95% O_2/_5% CO_2_. Coronal corticostriatal slices (300 μm thickness) were cut in this solution using a Leica VT1200S vibrating blade microtome (Leica Microsystems). For studies of evoked DA release, slices were maintained in HEPES-buffered aCSF at room temperature for 1 h before transfer to the recording chamber. For recording, slices were maintained at 32°C and superfused with recording aCSF at a flow rate of 1.5 mL/min controlled by a peristaltic pump (Gilson). The recording aCSF had the following composition (in mM): 124 NaCl, 3.7 KCl, 26 NaHCO_3_, 2.4 CaCl_2_, 1.3 MgSO_4_, 1.3 KH_2_PO_4_, and 10 glucose, equilibrated with 95% O_2_/5% CO_2_. Slices were allowed to acclimate to this environment for 30 min before recording was initiated^34,35,38,44^. Increases in extracellular DA concentration ([DA]_o_) were evoked by local electrical stimulation using a concentric stimulating electrode. Evoked increases in [DA]_o_ were quantified using 7-μm diameter carbon-fiber microelectrodes made in-house^107^ with a Millar voltammeter that generated a triangular FSCV waveform, −700 mV to +1300 mV then back to −700 mV *vs*. Ag/AgCl at a scan rate of 800 V/s, and recorded the resulting DA oxidation and reduction currents. Sampling frequency was 10 Hz; electrodes were out-of-circuit between scans to minimize DA adsorption^107^. Scans were initiated immediately after the electrode was placed in the superfusing aCSF and were repeated continuously under the control of a Master-8 timing circuit (AMPI). Data were collected using a Digidata 1550B controlled by AxoScope 10.7 software (Molecular Devices). A single stimulus pulse (100-ms duration, 0.4-mA amplitude) was sufficient to evoke DA release in the dlStr and NAc core, whereas a train of five pulses at 100 Hz was required to elicit reliable release in the NAc shell^34,42,44^. All electrodes were calibrated with a standard concentration of DA in aCSF in the recording chamber immediately after the last tissue measurement to indicate absolute evoked [DA]_o_^107^. The influence of exercise on DA release was assessed by comparing evoked [DA]_o_ between runners and controls. Two slices were examined from each animal, and evoked [DA]_o_ was recorded from 3-5 sites in the dlStr, in the NAc core, and in the NAc shell in each of these. This multiple-site sampling protocol is used to minimize sampling bias that can occur because site-to-site variability in the amplitude of evoked [DA]_o_ in a given region can exceed average differences between slices^34,38,42–44,108–110^. Initial sampling of evoked [DA]_o_ in two slices was completed in 20-30 min. Immediately afterward, superfusion with aCSF was either continued or the medium changed to aCSF plus a nAChR antagonist DHβE (1 µM) for 20 min, then recording of evoked release was repeated to test the possible involvement of cholinergic regulation of DA release via nAChRs^34,42–44^.

### Determination of *V*_max_ from evoked [DA]_o_ transients in striatal slices

To evaluate voluntary exercise-induced changes in DAT-mediated DA uptake, the initial portion of the falling phase of single pulse evoked [DA]_o_ curves was fitted to the Michaelis-Menten equation to extract *V*_max_ (maximal uptake rate)^108^. *K*_m_ (which is inversely related to the affinity of the DAT for DA) was fixed at 0.9 µM^43,108,111^. We evaluated *V*_max_ for dlStr and NAc core, but not for NAc shell because five-pulse stimulation rather than a single pulse was used in that region. Data with a goodness of fit R^2^ > 0.90 were included in the *V*_max_ analysis.

### Open field testing

Tests were conducted using an open-topped 40x40x40 cm acrylic arena with white walls and floor located in the center of the testing room. For testing, a mouse was removed from its home cage and placed in the center of the open field arena and allowed to explore freely for 60 minutes. The open field sessions were video recorded from above. Each animal’s movements were subsequently tracked, and movement patterns were analyzed using Noldus Ethovision XT 11.5. For analysis, each mouse was tracked based on three body landmarks (nose point, body centroid and tail), with the floor of the arena divided into two different zones: a central zone (defined as the area equivalent to 4 inner squares resulting from dividing the arena in 16 10×10 cm squares) and a border zone (10 cm from each of the walls) consisting of four sides and four corners. The outcome measures included the distance moved (m); mean velocity (cm/s); time spent moving (s); movement defined as change in coordinates of the center point of the mouse for >2 consecutive seconds; and frequency of entry, distance traveled and time spent within specific zones. Extracted measures were analyzed as the average across the entire 60-min period, as well as more granularly using consecutive 10-min bins.

### Vertical pole test

To assess motor coordination, a mouse was placed facing upwards at the top of a vertically-oriented, rough-surface plain steel rod (60 cm long and 1.2 cm in diameter), with the base of the pole placed in the center of the animal’s home cage. Each mouse was initially given two training trials to learn to turn and descend the pole back into the home cage. Each mouse was subsequently assessed across a video-recorded, 4-trial session; trials were limited to 120 s with a minimum interval of 15 min between trials. The time required to turn to orient downward and the total time to descend the pole were recorded. The mean and median scores across the 4 trials for each measure were used for analysis.

### Adhesive tape removal

For this test, a mouse was placed into an empty chamber of the same dimensions as its home cage and tested for adhesive tape removal from the forepaws to provide an index of possible changes in sensorimotor behavior. Small adhesive tape strips (0.3 cm × 0.4 cm) were applied on the forepaws of each animal at the same time so that they covered the hairless part of the paws. The time taken to make first contact with the tape, the paw used (left or right), the number of attempts, and the latency to remove the tape(s) were recorded. If the mouse did not contact or remove the tape(s) within 120 s, the trial was ended and the tape removed by the experimenter. Each mouse was given three adhesive tape removal trials, with a minimum interval of 15 mins between trials, and the results for each mouse averaged.

### Grip strength

Muscle/grip strength was assessed as the maximal horizontal force generated by the subject while grasping a 6 ×10 cm stainless steel grid platform connected to a sensitive force sensor (Bioseb)^112,113^. Two different grip strength indices were collected: forelimb only and combined forelimb and hindlimbs (all-limb). For the assessment of forelimb only grip strength, each mouse was removed from its home cage then given six testing trials with an inter-trial interval of 10-20 s. On each trial, the mouse was gently lowered onto the grid platform allowing only its forepaws to clasp onto the central top-half portion of the grid. Once both paws were grasping the grid, the mouse was pulled swiftly yet steadily away by the base of its tail which was held between the experimenter’s thumb and forefinger. Mice were pulled with the torso in a horizontal position until the grip was released from the grid. All-limb grip strength trials to assess the coordinated contribution of forelimbs and hindlimbs were performed similarly except that all four paws of the mouse were placed centrally on the grid and the torso of the mouse kept parallel to the grid during pulling. A 40-min interval was given between forelimb and all-limb measurements. The maximum force achieved for each trial was recorded as the peak tension (g) at the time the grasp was released for each trial. For all assessments, the truncated means of six consecutive trials (highest and lowest scores removed) were taken as the index of grip strength. Body weight was determined after grip strength testing was complete to evaluate for possible co-variability with performance.

### Statistics

All data are given as means ± SEM. Statistical analyses were conducted using Prism 10.4 (GraphPad Software, Inc.). For running activity, body weight measures, and food consumption, *n* = number of mice. Data were analyzed using 2-way ANOVA with repeated measures or with a mixed effects model using sex or exercise as between-subjects factor and timepoint as within-subjects factor (Supplementary Table 1). Significant interactions were decomposed with Sidák’s multiple comparisons *post hoc* test. For FSCV data, *n* = number of recording sites from multiple-site sampling for each cohort of runners or controls. For each data set, normality was determined using D’Agostino-Pearson test (α = 0.05 for normality), and equal variance was assessed using F-tests. The influence of exercise on evoked [DA]_o_ and *V*_max_ in a given brain region and given sex was assessed using an unpaired Student’s *t*-test (parametric) with or without Welch’s correction, or Mann-Whitney *U*-test (nonparametric) as appropriate for the data. Velocity in the open field by time bin was analyzed by 2-way ANOVA with repeated measures and Sidák’s multiple comparison *post hoc* test (Supplementary Table 2). Data for all other parameters in the open field, as well as other motor performance tests were analyzed using 2-way ANOVA with sex and exercise as between-subject factors and Sidák’s multiple comparisons of selected pairs *post hoc* test. When data analysis did not show sex differences between controls or runners, data for both sexes were combined and unpaired Student’s *t*-tests were used (Supplementary Table 2). It should be noted that in some cases, the general 2-way ANOVA indicated a sex difference, but no significant differences were seen between relevant pairs in *post hoc* comparisons (see Supplementary Table 2). Differences were considered significant when *p* < 0.05.

## Supplementary information

Supplementary Information

## Data Availability

All data used in this study are available upon request to the corresponding author.

## References

[1] Alexander, G. E. et al. Characterizing cognitive aging in humans with links to animal models. *Front Aging Neurosci.* **4**, 21 (2012). 22988439 10.3389/fnagi.2012.00021 PMC3439638

[2] Martini, D. N. et al. Exploring the effects of dopamine on sensorimotor inhibition and mobility in older adults. *Exp. Brain Res* **241**, 127–133 (2023). 36394592 10.1007/s00221-022-06509-1 PMC9870938

[3] Moskowitz, S. et al. Is impaired dopaminergic function associated with mobility capacity in older adults?. *Geroscience* **43**, 1383–1404 (2021). 33236263 10.1007/s11357-020-00303-z PMC8190430

[4] Coleman, C. R. et al. Natural variation in age-related dopamine neuron degeneration is glutathione dependent and linked to life span. *Proc. Natl Acad. Sci. USA* **121**, e2403450121 (2024). 39388265 10.1073/pnas.2403450121 PMC11494315

[5] Calabresi, P., Galletti, F., Saggese, E., Ghiglieri, V. & Picconi, B. Neuronal networks and synaptic plasticity in Parkinson’s disease: beyond motor deficits. *Parkinsonism Relat. Disord.* **13**, S259–S262 (2007). 18267247 10.1016/S1353-8020(08)70013-0

[6] Schultz, W. Predictive reward signal of dopamine neurons. *J. Neurophysiol.* **80**, 1–27 (1998). 9658025 10.1152/jn.1998.80.1.1

[7] Matsuda, W. et al. Single nigrostriatal dopaminergic neurons form widely spread and highly dense axonal arborizations in the neostriatum. *J. Neurosci.* **29**, 444–453 (2009). 19144844 10.1523/JNEUROSCI.4029-08.2009 PMC6664950

[8] Rice, M. E., Patel, J. C. & Cragg, S. J. Dopamine release in the basal ganglia. *Neuroscience* **198**, 112–137 (2011). 21939738 10.1016/j.neuroscience.2011.08.066 PMC3357127

[9] Sulzer, D., Cragg, S. J. & Rice, M. E. Striatal dopamine neurotransmission: regulation of release and uptake. *Basal Ganglia* **6**, 123–148 (2016). 27141430 10.1016/j.baga.2016.02.001 PMC4850498

[10] Fearnley, J. M. & Lees, A. J. Ageing and Parkinson’s disease: substantia nigra regional selectivity. *Brain* **114**, 2283–2301 (1991). 1933245 10.1093/brain/114.5.2283

[11] Pineda-Pardo, J. A., Sanchez-Ferro, A., Monje, M. H. G., Pavese, N. & Obeso, J. A. Onset pattern of nigrostriatal denervation in early Parkinson’s disease. *Brain* **145**, 1018–1028 (2022). 35349639 10.1093/brain/awab378 PMC9351472

[12] Kaasinen, V. & Rinne, J. O. Functional imaging studies of dopamine system and cognition in normal aging and Parkinson’s disease. *Neurosci. Biobehav Rev.* **26**, 785–793 (2002). 12470690 10.1016/s0149-7634(02)00065-9

[13] Panigrahi, B. et al. Dopamine Is Required for the Neural Representation and Control of Movement Vigor. *Cell* **162**, 1418–1430 (2015). 26359992 10.1016/j.cell.2015.08.014

[14] Vivar, C. & van Praag, H. Running Changes the Brain: the Long and the Short of It. *Physiol. (Bethesda)* **32**, 410–424 (2017). 10.1152/physiol.00017.2017 PMC6148340 29021361

[15] Sujkowski, A., Hong, L., Wessells, R. J. & Todi, S. V. The protective role of exercise against age-related neurodegeneration. *Ageing Res Rev.* **74**, 101543 (2022). 34923167 10.1016/j.arr.2021.101543 PMC8761166

[16] Jia, Y. et al. Aerobic Physical Exercise as a non-medical intervention for brain dysfunction: state of the art and beyond. *Front Neurol.* **13**, 862078 (2022). 35645958 10.3389/fneur.2022.862078 PMC9136296

[17] Pourteymour, S., Majhi, R. K., Norheim, F. A. & Drevon, C. A. Exercise delays brain ageing through muscle-brain crosstalk. *Cell Prolif.* **58**, e70026 (2025). 40125692 10.1111/cpr.70026 PMC12240646

[18] Vivar, C., Potter, M. C. & van Praag, H. All about running: synaptic plasticity, growth factors and adult hippocampal neurogenesis. *Curr. Top. Behav. Neurosci.* **15**, 189–210 (2013). 22847651 10.1007/7854_2012_220 PMC4565722

[19] Erickson, K. I. et al. Exercise training increases size of hippocampus and improves memory. *Proc. Natl Acad. Sci. USA* **108**, 3017–3022 (2011). 21282661 10.1073/pnas.1015950108 PMC3041121

[20] Tillerson, J. L., Caudle, W. M., Reveron, M. E. & Miller, G. W. Exercise induces behavioral recovery and attenuates neurochemical deficits in rodent models of Parkinson’s disease. *Neuroscience* **119**, 899–911 (2003). 12809709 10.1016/s0306-4522(03)00096-4

[21] Petzinger, G. M. et al. Effects of treadmill exercise on dopaminergic transmission in the 1-methyl-4-phenyl-1,2,3,6-tetrahydropyridine-lesioned mouse model of basal ganglia injury. *J. Neurosci.* **27**, 5291–5300 (2007). 17507552 10.1523/JNEUROSCI.1069-07.2007 PMC6672356

[22] Tajiri, N. et al. Exercise exerts neuroprotective effects on Parkinson’s disease model of rats. *Brain Res* **1310**, 200–207 (2010). 19900418 10.1016/j.brainres.2009.10.075

[23] Petzinger, G. M. et al. Exercise-enhanced neuroplasticity targeting motor and cognitive circuitry in Parkinson’s disease. *Lancet Neurol.* **12**, 716–726 (2013). 23769598 10.1016/S1474-4422(13)70123-6 PMC3690528

[24] Regensburger, M., Prots, I. & Winner, B. Adult hippocampal neurogenesis in Parkinson’s disease: impact on neuronal survival and plasticity. *Neural Plast.* **2014**, 454696 (2014). 25110593 10.1155/2014/454696 PMC4106176

[25] Zigmond, M. J. & Smeyne, R. J. Exercise: is it a neuroprotective and if so, how does it work?. *Parkinsonism Relat. Disord.* **20**, S123–S127 (2014). 24262162 10.1016/S1353-8020(13)70030-0

[26] Churchill, M. J. et al. Exercise in an animal model of Parkinson’s disease: Motor recovery but not restoration of the nigrostriatal pathway. *Neuroscience* **359**, 224–247 (2017). 28754312 10.1016/j.neuroscience.2017.07.031

[27] Hou, L., Chen, W., Liu, X., Qiao, D. & Zhou, F. M. Exercise-induced neuroprotection of the nigrostriatal dopamine system in Parkinson’s disease. *Front Aging Neurosci.* **9**, 358 (2017). 29163139 10.3389/fnagi.2017.00358 PMC5675869

[28] Chen, Y. H. et al. Exercise ameliorates motor deficits and improves dopaminergic functions in the rat hemi-Parkinson’s model. *Sci. Rep.* **8**, 3973 (2018). 29507426 10.1038/s41598-018-22462-y PMC5838260

[29] Wang, X. et al. Aerobic exercise improves motor function and striatal MSNs-Erk/MAPK signaling in mice with 6-OHDA-induced Parkinson’s disease. *Exp. Brain Res* **240**, 1713–1725 (2022). 35384454 10.1007/s00221-022-06360-4 PMC8985567

[30] Fox, S. H. et al. The Movement disorder society evidence-based medicine review update: Treatments for the motor symptoms of Parkinson’s disease. *Mov. Disord.* **26**, S2–S41 (2011). 22021173 10.1002/mds.23829

[31] Shen, X., Wong-Yu, I. S. & Mak, M. K. Effects of exercise on falls, balance, and gait ability in Parkinson’s disease: A Meta-analysis. *Neurorehabil Neural Repair* **30**, 512–527 (2016). 26493731 10.1177/1545968315613447

[32] Lee, J., Choi, M. & Yoo, Y. A Meta-analysis of nonpharmacological interventions for people with Parkinson’s disease. *Clin. Nurs. Res* **26**, 608–631 (2017). 27318243 10.1177/1054773816655091

[33] Luthra, N. S. et al. Aerobic exercise-induced changes in fluid biomarkers in Parkinson’s disease. *NPJ Parkinsons Dis.* **11**, 190 (2025). 40595707 10.1038/s41531-025-01042-8 PMC12215721

[34] Bastioli, G. et al. Voluntary exercise boosts striatal dopamine release: evidence for the necessary and sufficient role of BDNF. *J. Neurosci.* **42**, 4725–4736 (2022). 35577554 10.1523/JNEUROSCI.2273-21.2022 PMC9186798

[35] Rice, M. E. & Cragg, S. J. Nicotine amplifies reward-related dopamine signals in striatum. *Nat. Neurosci.* **7**, 583–584 (2004). 15146188 10.1038/nn1244

[36] Zhang, H. & Sulzer, D. Frequency-dependent modulation of dopamine release by nicotine. *Nat. Neurosci.* **7**, 581–582 (2004). 15146187 10.1038/nn1243

[37] Cachope, R. et al. Selective activation of cholinergic interneurons enhances accumbal phasic dopamine release: setting the tone for reward processing. *Cell Rep.* **2**, 33–41 (2012). 22840394 10.1016/j.celrep.2012.05.011 PMC3408582

[38] Patel, J. C., Rossignol, E., Rice, M. E. & Machold, R. P. Opposing regulation of dopaminergic activity and exploratory motor behavior by forebrain and brainstem cholinergic circuits. *Nat. Commun.* **3**, 1172 (2012). 23132022 10.1038/ncomms2144 PMC5336695

[39] Threlfell, S. et al. Striatal dopamine release is triggered by synchronized activity in cholinergic interneurons. *Neuron* **75**, 58–64 (2012). 22794260 10.1016/j.neuron.2012.04.038

[40] Kramer, P. F. et al. Synaptic-like axo-axonal transmission from striatal cholinergic interneurons onto dopaminergic fibers. *Neuron* **110**, 2949–2960.e2944 (2022). 35931070 10.1016/j.neuron.2022.07.011 PMC9509469

[41] Liu, C. et al. An action potential initiation mechanism in distal axons for the control of dopamine release. *Science* **375**, 1378–1385 (2022). 35324301 10.1126/science.abn0532 PMC9081985

[42] Stouffer, M. A. et al. Insulin enhances striatal dopamine release by activating cholinergic interneurons and thereby signals reward. *Nat. Commun.* **6**, 8543 (2015). 26503322 10.1038/ncomms9543 PMC4624275

[43] Patel, J. C. et al. Interactions between insulin and diet on striatal dopamine uptake kinetics in rodent brain slices. *Eur. J. Neurosci.* **49**, 794–804 (2019). 29791756 10.1111/ejn.13958 PMC6613817

[44] Mancini, M., Patel, J. C., Affinati, A. H., Witkovsky, P. & Rice, M. E. Leptin promotes striatal dopamine release via cholinergic interneurons and regionally distinct signaling pathways. *J. Neurosci.* **42**, 6668–6679 (2022). 35906070 10.1523/JNEUROSCI.0238-22.2022 PMC9436012

[45] Flint, A. The source of muscular power, as deduced from observations upon the human subject under conditions of rest and of exercise. *J. Anat. Physiol.* **12**, 91–141 (1877). 17231187 PMC1309827

[46] Fiuza-Luces, C. et al. Exercise benefits in cardiovascular disease: beyond attenuation of traditional risk factors. *Nat. Rev. Cardiol.* **15**, 731–743 (2018). 30115967 10.1038/s41569-018-0065-1

[47] Isath, A. et al. Exercise and cardiovascular health: A state-of-the-art review. *Prog. Cardiovasc Dis.* **79**, 44–52 (2023). 37120119 10.1016/j.pcad.2023.04.008

[48] Chen, C. & Nakagawa, S. Physical activity for cognitive health promotion: An overview of the underlying neurobiological mechanisms. *Ageing Res Rev.* **86**, 101868 (2023). 36736379 10.1016/j.arr.2023.101868

[49] De la Rosa, A. et al. Physical exercise in the prevention and treatment of Alzheimer’s disease. *J. Sport Health Sci.* **9**, 394–404 (2020). 32780691 10.1016/j.jshs.2020.01.004 PMC7498620

[50] Zikereya, T., Shi, K. & Chen, W. Goal-directed and habitual control: from circuits and functions to exercise-induced neuroplasticity targets for the treatment of Parkinson’s disease. *Front Neurol.* **14**, 1254447 (2023). 37881310 10.3389/fneur.2023.1254447 PMC10597699

[51] Manzanares, G., Brito-da-Silva, G. & Gandra, P. G. Voluntary wheel running: patterns and physiological effects in mice. *Braz. J. Med Biol. Res* **52**, e7830 (2018). 30539969 10.1590/1414-431X20187830 PMC6301263

[52] Tanner, M. K. et al. Duration- and sex-dependent neural circuit control of voluntary physical activity. *Psychopharmacol. (Berl.)* **239**, 3697–3709 (2022). 10.1007/s00213-022-06243-0 PMC9768838 36195731

[53] Lightfoot, J. T., Turner, M. J., Daves, M., Vordermark, A. & Kleeberger, S. R. Genetic influence on daily wheel running activity level. *Physiol. Genomics* **19**, 270–276 (2004). 15383638 10.1152/physiolgenomics.00125.2004

[54] Dworatzek, E. et al. Sex differences in exercise-induced physiological myocardial hypertrophy are modulated by oestrogen receptor beta. *Cardiovasc Res* **102**, 418–428 (2014). 24654233 10.1093/cvr/cvu065

[55] Mathis, V. et al. Estrogen-mediated individual differences in female rat voluntary running behavior. *J. Appl Physiol.* **136**, 592–605 (2024). 38299221 10.1152/japplphysiol.00611.2023 PMC11212800

[56] Bartling, B. et al. Sex-related differences in the wheel-running activity of mice decline with increasing age. *Exp. Gerontol.* **87**, 139–147 (2017). 27108181 10.1016/j.exger.2016.04.011

[57] Nelson, J. F., Felicio, L. S., Randall, P. K., Sims, C. & Finch, C. E. A longitudinal study of estrous cyclicity in aging C57BL/6J mice: I. Cycle frequency, length and vaginal cytology. *Biol. Reprod.* **27**, 327–339 (1982). 6889895 10.1095/biolreprod27.2.327

[58] Schulz, H. et al. Respiratory mechanics in mice: strain and sex specific differences. *Acta Physiol. Scand.* **174**, 367–375 (2002). 11942924 10.1046/j.1365-201x.2002.00955.x

[59] Palmer, L. A. et al. Hypoxia-induced ventilatory responses in conscious mice: gender differences in ventilatory roll-off and facilitation. *Respir. Physiol. Neurobiol.* **185**, 497–505 (2013). 23183420 10.1016/j.resp.2012.11.010 PMC3593587

[60] Novak, C. M., Burghardt, P. R. & Levine, J. A. The use of a running wheel to measure activity in rodents: relationship to energy balance, general activity, and reward. *Neurosci. Biobehav Rev.* **36**, 1001–1014 (2012). 22230703 10.1016/j.neubiorev.2011.12.012 PMC4455940

[61] Lu, B. Acute and long-term synaptic modulation by neurotrophins. *Prog. Brain Res* **146**, 137–150 (2004). 14699962 10.1016/s0079-6123(03)46010-x

[62] Lu, B., Nagappan, G., Guan, X., Nathan, P. J. & Wren, P. BDNF-based synaptic repair as a disease-modifying strategy for neurodegenerative diseases. *Nat. Rev. Neurosci.* **14**, 401–416 (2013). 23674053 10.1038/nrn3505

[63] Park, H. & Poo, M. M. Neurotrophin regulation of neural circuit development and function. *Nat. Rev. Neurosci.* **14**, 7–23 (2013). 23254191 10.1038/nrn3379

[64] Goggi, J., Pullar, I. A., Carney, S. L. & Bradford, H. F. Modulation of neurotransmitter release induced by brain-derived neurotrophic factor in rat brain striatal slices in vitro. *Brain Res* **941**, 34–42 (2002). 12031545 10.1016/s0006-8993(02)02505-2

[65] Goggi, J., Pullar, I. A., Carney, S. L. & Bradford, H. F. Signalling pathways involved in the short-term potentiation of dopamine release by BDNF. *Brain Res* **968**, 156–161 (2003). 12644273 10.1016/s0006-8993(03)02234-0

[66] Bosse, K. E. et al. Aberrant striatal dopamine transmitter dynamics in brain-derived neurotrophic factor-deficient mice. *J. Neurochem* **120**, 385–395 (2012). 21988371 10.1111/j.1471-4159.2011.07531.x PMC3385875

[67] Mogi, M. et al. Brain-derived growth factor and nerve growth factor concentrations are decreased in the substantia nigra in Parkinson’s disease. *Neurosci. Lett.* **270**, 45–48 (1999). 10454142 10.1016/s0304-3940(99)00463-2

[68] Parain, K. et al. Reduced expression of brain-derived neurotrophic factor protein in Parkinson’s disease substantia nigra. *Neuroreport* **10**, 557–561 (1999). 10208589 10.1097/00001756-199902250-00021

[69] Howells, D. W. et al. Reduced BDNF mRNA expression in the Parkinson’s disease substantia nigra. *Exp. Neurol.* **166**, 127–135 (2000). 11031089 10.1006/exnr.2000.7483

[70] Gerecke, K. M., Jiao, Y., Pani, A., Pagala, V. & Smeyne, R. J. Exercise protects against MPTP-induced neurotoxicity in mice. *Brain Res* **1341**, 72–83 (2010). 20116369 10.1016/j.brainres.2010.01.053 PMC2884060

[71] Gerecke, K. M., Jiao, Y., Pagala, V. & Smeyne, R. J. Exercise does not protect against MPTP-induced neurotoxicity in BDNF haploinsufficient mice. *PLoS One* **7**, e43250 (2012). 22912838 10.1371/journal.pone.0043250 PMC3422268

[72] Ahlskog, J. E. Does vigorous exercise have a neuroprotective effect in Parkinson disease?. *Neurology* **77**, 288–294 (2011). 21768599 10.1212/WNL.0b013e318225ab66 PMC3136051

[73] Ahlskog, J. E. Aerobic Exercise: Evidence for a direct brain effect to slow Parkinson disease progression. *Mayo Clin. Proc.* **93**, 360–372 (2018). 29502566 10.1016/j.mayocp.2017.12.015

[74] Voss, M. W. et al. The influence of aerobic fitness on cerebral white matter integrity and cognitive function in older adults: results of a one-year exercise intervention. *Hum. Brain Mapp.* **34**, 2972–2985 (2013). 22674729 10.1002/hbm.22119 PMC4096122

[75] Shulman, L. M. et al. Randomized clinical trial of 3 types of physical exercise for patients with Parkinson disease. *JAMA Neurol.* **70**, 183–190 (2013). 23128427 10.1001/jamaneurol.2013.646 PMC4574905

[76] Heumann, R. et al. Dyskinesia in Parkinson’s disease: mechanisms and current non-pharmacological interventions. *J. Neurochem* **130**, 472–489 (2014). 24773031 10.1111/jnc.12751

[77] Frazzitta, G. et al. Intensive rehabilitation treatment in early Parkinson’s disease: a randomized pilot study with a 2-year follow-up. *Neurorehabil Neural Repair* **29**, 123–131 (2015). 25038064 10.1177/1545968314542981

[78] Duchesne, C. et al. Enhancing both motor and cognitive functioning in Parkinson’s disease: Aerobic exercise as a rehabilitative intervention. *Brain Cogn.* **99**, 68–77 (2015). 26263381 10.1016/j.bandc.2015.07.005

[79] Monteiro-Junior, R. S. et al. We need to move more: Neurobiological hypotheses of physical exercise as a treatment for Parkinson’s disease. *Med Hypotheses* **85**, 537–541 (2015). 26209418 10.1016/j.mehy.2015.07.011

[80] Jakowec, M. W., Wang, Z., Holschneider, D., Beeler, J. & Petzinger, G. M. Engaging cognitive circuits to promote motor recovery in degenerative disorders. exercise as a learning modality. *J. Hum. Kinet.* **52**, 35–51 (2016). 28149392 10.1515/hukin-2015-0192 PMC5260516

[81] LaHue, S. C., Comella, C. L. & Tanner, C. M. The best medicine? The influence of physical activity and inactivity on Parkinson’s disease. *Mov. Disord.* **31**, 1444–1454 (2016). 27477046 10.1002/mds.26728 PMC9491025

[82] da Silva, P. G., Domingues, D. D., de Carvalho, L. A., Allodi, S. & Correa, C. L. Neurotrophic factors in Parkinson’s disease are regulated by exercise: Evidence-based practice. *J. Neurol. Sci.* **363**, 5–15 (2016). 27000212 10.1016/j.jns.2016.02.017

[83] Schootemeijer, S., van der Kolk, N. M., Bloem, B. R. & de Vries, N. M. Current perspectives on aerobic exercise in people with Parkinson’s disease. *Neurotherapeutics* **17**, 1418–1433 (2020). 32808252 10.1007/s13311-020-00904-8 PMC7851311

[84] Wu, S. Y. et al. Running exercise protects the substantia nigra dopaminergic neurons against inflammation-induced degeneration via the activation of BDNF signaling pathway. *Brain Behav. Immun.* **25**, 135–146 (2011). 20851176 10.1016/j.bbi.2010.09.006

[85] Zhou, W., Barkow, J. C. & Freed, C. R. Running wheel exercise reduces alpha-synuclein aggregation and improves motor and cognitive function in a transgenic mouse model of Parkinson’s disease. *PLoS One* **12**, e0190160 (2017). 29272304 10.1371/journal.pone.0190160 PMC5741244

[86] Neeper, S. A., Gomez-Pinilla, F., Choi, J. & Cotman, C. Exercise and brain neurotrophins. *Nature* **373**, 109 (1995). 7816089 10.1038/373109a0

[87] van Praag, H., Christie, B. R., Sejnowski, T. J. & Gage, F. H. Running enhances neurogenesis, learning, and long-term potentiation in mice. *Proc. Natl Acad. Sci. USA* **96**, 13427–13431 (1999). 10557337 10.1073/pnas.96.23.13427 PMC23964

[88] Cotman, C. W., Berchtold, N. C. & Christie, L. A. Exercise builds brain health: key roles of growth factor cascades and inflammation. *Trends Neurosci.* **30**, 464–472 (2007). 17765329 10.1016/j.tins.2007.06.011

[89] Sleiman, S. F. & Chao, M. V. Downstream consequences of exercise through the action of BDNF. *Brain Plast.* **1**, 143–148 (2015). 29765838 10.3233/BPL-150017 PMC5939187

[90] Choi, S. H. et al. Combined adult neurogenesis and BDNF mimic exercise effects on cognition in an Alzheimer’s mouse model. *Science* **361**, 10.1126/science.aan8821 (2018). 10.1126/science.aan8821 PMC6149542 30190379

[91] Tomlinson, C. L. et al. Physiotherapy versus placebo or no intervention in Parkinson’s disease. *Cochrane Database Syst. Rev.* **2013**, CD002817 (2013). 22786482 10.1002/14651858.CD002817.pub2

[92] Carlsson, A. Treatment of Parkinson’s with L-DOPA. The early discovery phase, and a comment on current problems. *J. Neural Transm. (Vienna)* **109**, 777–787 (2002). 12111467 10.1007/s007020200064

[93] Carta, M. & Bezard, E. Contribution of pre-synaptic mechanisms to L-DOPA-induced dyskinesia. *Neuroscience* **198**, 245–251 (2011). 21840375 10.1016/j.neuroscience.2011.07.070

[94] Girasole, A. E. et al. A Subpopulation of striatal neurons mediates levodopa-induced dyskinesia. *Neuron* **97**, 787–795.e786 (2018). 29398356 10.1016/j.neuron.2018.01.017 PMC6233726

[95] Ryan, M. B., Bair-Marshall, C. & Nelson, A. B. Aberrant striatal activity in Parkinsonism and levodopa-induced dyskinesia. *Cell Rep.* **23**, 3438–3446.e3435 (2018). 29924988 10.1016/j.celrep.2018.05.059 PMC6407866

[96] Aguiar, A. S. Jr. et al. Short bouts of mild-intensity physical exercise improve spatial learning and memory in aging rats: involvement of hippocampal plasticity via AKT, CREB and BDNF signaling. *Mech. Ageing Dev.* **132**, 560–567 (2011). 21983475 10.1016/j.mad.2011.09.005

[97] Cotman, C. W. & Berchtold, N. C. Exercise: a behavioral intervention to enhance brain health and plasticity. *Trends Neurosci.* **25**, 295–301 (2002). 12086747 10.1016/s0166-2236(02)02143-4

[98] Gomez-Pinilla, F., So, V. & Kesslak, J. P. Spatial learning and physical activity contribute to the induction of fibroblast growth factor: neural substrates for increased cognition associated with exercise. *Neuroscience* **85**, 53–61 (1998). 9607702 10.1016/s0306-4522(97)00576-9

[99] Barnes, C. A. et al. Exercise does not modify spatial memory, brain autoimmunity, or antibody response in aged F-344 rats. *Neurobiol. Aging* **12**, 47–53 (1991). 2002883 10.1016/0197-4580(91)90038-l

[100] Gorrell, S., Shott, M. E. & Frank, G. K. W. Associations between aerobic exercise and dopamine-related reward-processing: Informing a model of human exercise engagement. *Biol. Psychol.* **171**, 108350 (2022). 35561818 10.1016/j.biopsycho.2022.108350 PMC9869713

[101] Ferrazzoli, D. et al. The ties that bind: Aberrant plasticity and networks dysfunction in movement disorders-implications for rehabilitation. *Brain Connect* **11**, 278–296 (2021). 33403893 10.1089/brain.2020.0971

[102] Wang, Y. et al. Aerobic exercise improves motor dysfunction in Parkinson’s model mice via differential regulation of striatal medium spiny neuron. *Sci. Rep.* **14**, 12132 (2024). 38802497 10.1038/s41598-024-63045-4 PMC11130133

[103] Cheung, T. H. C., Ding, Y., Zhuang, X. & Kang, U. J. Learning critically drives parkinsonian motor deficits through imbalanced striatal pathway recruitment. *Proc. Natl Acad. Sci. USA* **120**, e2213093120 (2023). 36920928 10.1073/pnas.2213093120 PMC10041136

[104] Hart, G., Burton, T. J. & Balleine, B. W. What role does striatal dopamine play in goal-directed action?. *Neuroscience* **546**, 20–32 (2024). 38521480 10.1016/j.neuroscience.2024.03.020

[105] Lee, J. & Sabatini, B. L. From avoidance to new action: the multifaceted role of the striatal indirect pathway. *Nat. Rev. Neurosci.* **26**, 438–449 (2025). 40335770 10.1038/s41583-025-00925-2

[106] Sleiman, S. F. et al. Exercise promotes the expression of brain derived neurotrophic factor (BDNF) through the action of the ketone body beta-hydroxybutyrate. *Elife* **5**, 10.7554/eLife.15092 (2016). 10.7554/eLife.15092 PMC4915811 27253067

[107] Patel, J. C. & Rice, M. E. Monitoring axonal and somatodendritic dopamine release using fast-scan cyclic voltammetry in brain slices. *Methods Mol. Biol.* **964**, 243–273 (2013). 23296788 10.1007/978-1-62703-251-3_15

[108] Li, X. et al. Enhanced striatal dopamine transmission and motor performance with LRRK2 overexpression in mice is eliminated by familial Parkinson’s disease mutation G2019S. *J. Neurosci.* **30**, 1788–1797 (2010). 20130188 10.1523/JNEUROSCI.5604-09.2010 PMC2858426

[109] Karayannis, T. et al. Cntnap4 differentially contributes to GABAergic and dopaminergic synaptic transmission. *Nature* **511**, 236–240 (2014). 24870235 10.1038/nature13248 PMC4281262

[110] Longo, F. et al. Cell-type-specific disruption of PERK-eIF2alpha signaling in dopaminergic neurons alters motor and cognitive function. *Mol. Psychiatry* **26**, 6427–6450 (2021). 33879865 10.1038/s41380-021-01099-w PMC8526653

[111] Wu, Q., Reith, M. E., Wightman, R. M., Kawagoe, K. T. & Garris, P. A. Determination of release and uptake parameters from electrically evoked dopamine dynamics measured by real-time voltammetry. *J. Neurosci. Methods* **112**, 119–133 (2001). 11716947 10.1016/s0165-0270(01)00459-9

[112] Cabe, P. A., Tilson, H. A., Mitchell, C. L. & Dennis, R. A simple recording grip strength device. *Pharm. Biochem Behav.* **8**, 101–102 (1978). 10.1016/0091-3057(78)90131-4 625478

[113] Ge, X. et al. Grip strength is potentially an early indicator of age-related decline in mice. *Pathobiol. Aging Age Relat. Dis.* **6**, 32981 (2016). 27613499 10.3402/pba.v6.32981 PMC5018066

