# Supplementary material for: Voluntary exercise increases striatal dopamine release and improves motor performance in aging mice
Source: NPJ Parkinsons Dis. 2025 Dec 9;11:345. doi: 10.1038/s41531-025-01213-7 (PMC12690118; doi:10.1038/s41531-025-01213-7)
Supplement: Supplementary file 1 — Supplementary Information [file 41531_2025_1213_MOESM1_ESM.pdf]

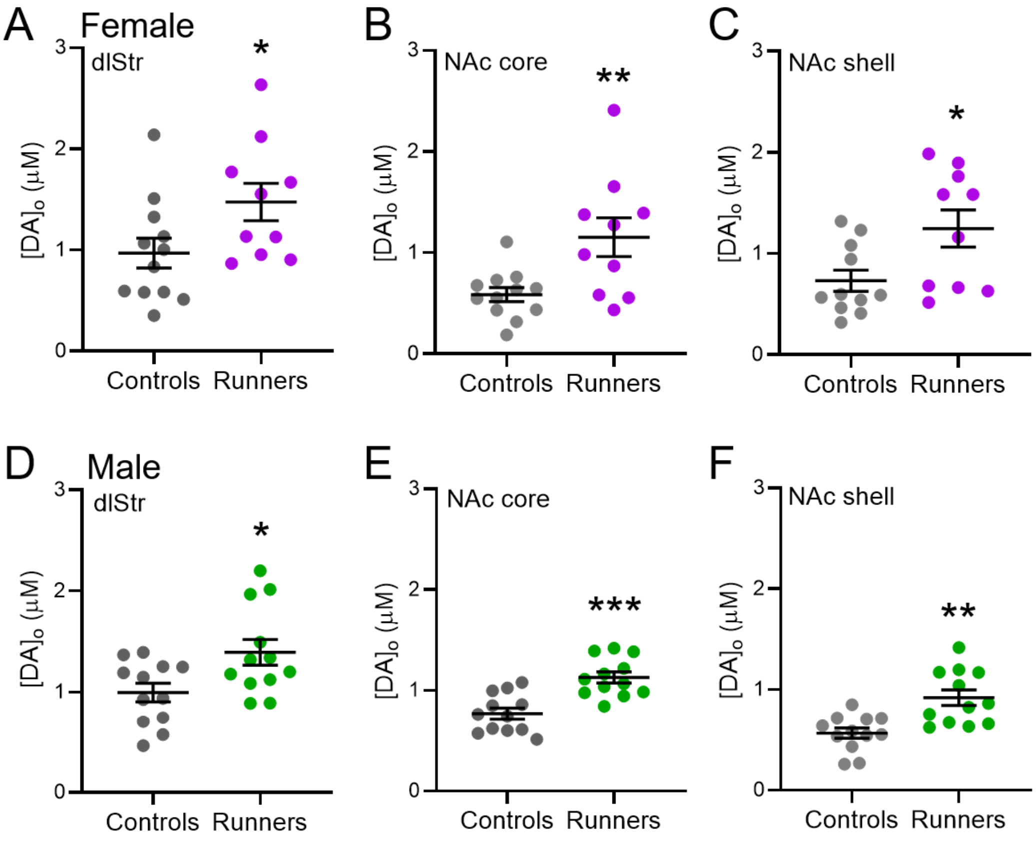

Supplemental Fig. 1. **Increased evoked $[DA]_o$ after 30 d of voluntary wheel running in striatal slices from female and from male mice, averaged per slice. A-C.** Mean evoked $[DA]_o$ in dStr, and NAc core and shell from female runners and controls (*$p < 0.05$, **$p < 0.01$, 2 slices/mouse, 6 mice/group; unpaired *t*-test). **D-F.** Mean evoked $[DA]_o$ in dStr, and NAc core and shell from male runners and controls (*$p < 0.05$, **$p < 0.01$, ***$p < 0.001$, 2 slices/mouse, 6 mice/group; unpaired *t*-tests).

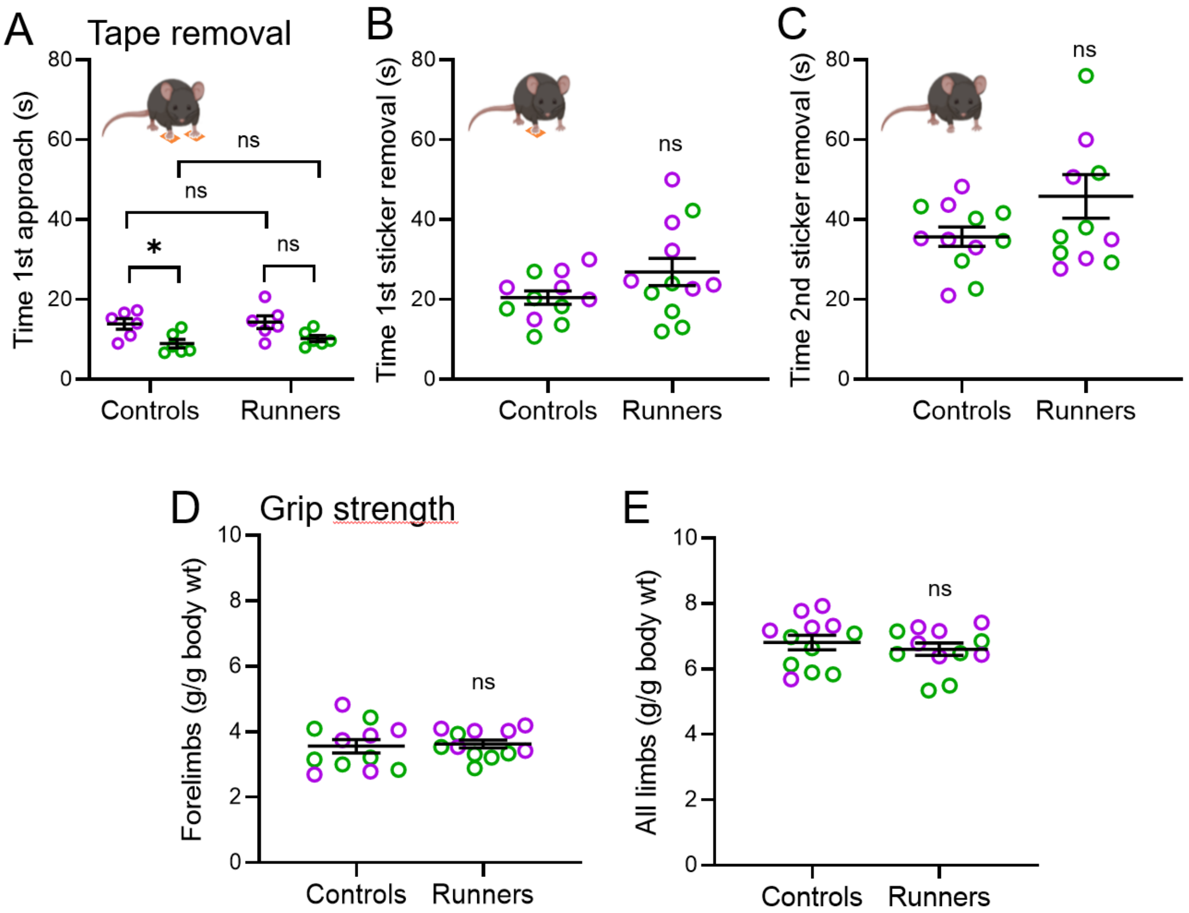

Supplemental Fig. 2. Adhesive removal and grip strength tests. A-C. Adhesive tape removal; insets show tape placements on forepaws. **A.** Latency until first approach (ns not significant, *$p < 0.05$, n = 6 per group for each sex; 2-way ANOVA with Sidák's *post hoc* test). **B.** Latency to remove first tape (ns not significant, runners *vs*. controls; n = 12 per group, sexes pooled; unpaired *t*-test). **C.** Latency to remove second tape (ns not significant, runners *vs*. controls; n = 12 per group, sexes pooled; unpaired *t*-test). **F,G.** Grip strength. **F.** Forelimb grip strength (ns not significant, runners *vs*. controls; n = 12 per group, sexes pooled; unpaired *t*-test). **G.** Combined forelimb and hindlimb grip strength (ns not significant, runners *vs*. controls; sexes pooled; unpaired *t*-test).

Supplemental Table 1: Statistical Analysis for Wheel Running, Body Weight and Food Consumption				
	2-way ANOVA (repeated measures / mixed model effects)		*Post hoc* test	
	**F(DFn, DFd)**	***p* value**	**Sidák's multiple comparisons**	***p* value**
**Wheel Running**				
**Average Daily Running (rev/h)**				
Time	F(3.048,1125) = 536.9	<0.0001 (***)	ZT 1: Female *vs.* Male<br>ZT 2: Female *vs.* Male<br>ZT 3: Female *vs.* Male<br>ZT 4: Female *vs.* Male<br>ZT 5: Female *vs.* Male<br>ZT 6: Female *vs.* Male<br>ZT 7: Female *vs.* Male<br>ZT 8: Female *vs.* Male<br>ZT 9: Female *vs.* Male<br>ZT 10: Female *vs.* Male<br>ZT 11: Female *vs.* Male<br>ZT 12-24: Female *vs.* Male	0.0003 (***)<br>0.4126 (ns)<br>>0.9999 (ns)<br>0.2867 (ns)<br>0.0488 (*)<br>0.0304 (*)<br>0.4895 (ns)<br>0.0040 (**)<br>0.0439 (*)<br>0.6339 (ns)<br>>0.9999 (ns)<br><0.0001 (***)
Sex	F(1,369) = 293.5	<0.0001 (***)		
Interaction	F(23,8487) = 130.4	<0.0001 (***)		
Subject	F(369,8487) = 8.250	<0.0001 (***)		
**Total Running Activity (rev/day)**				
Time	F(3.053,30.53) = 28.87	<0.0001 (***)	Day 1: Female *vs.* Male<br>Day 2: Female *vs.* Male<br>Day 3: Female *vs.* Male<br>Day 4: Female *vs.* Male<br>Day 5: Female *vs.* Male<br>Day 6: Female *vs.* Male<br>Day 7: Female *vs.* Male<br>Day 8: Female *vs.* Male<br>Day 9: Female *vs.* Male<br>Day 10: Female *vs.* Male<br>Day 11: Female *vs.* Male<br>Day 12: Female *vs.* Male<br>Day 13: Female *vs.* Male<br>Day 14: Female *vs.* Male<br>Day 15: Female *vs.* Male<br>Day 16: Female *vs.* Male<br>Day 17: Female *vs.* Male<br>Day 18: Female *vs.* Male<br>Day 19: Female *vs.* Male<br>Day 20: Female *vs.* Male<br>Day 21: Female *vs.* Male<br>Day 22: Female *vs.* Male<br>Day 23: Female *vs.* Male<br>Day 24: Female *vs.* Male<br>Day 25: Female *vs.* Male<br>Day 26: Female *vs.* Male	0.7470 (ns)<br>0.4389 (ns)<br>0.1327 (ns)<br>0.0264 (*)<br>0.0148 (*)<br>0.0055 (**)<br>0.0637 (ns)<br>0.0041 (**)<br>0.0180 (*)<br>0.0617 (ns)<br>0.0716 (ns)<br>0.0260 (*)<br>0.0643 (ns)<br>0.0334 (*)<br>0.0357 (*)<br>0.0232 (*)<br>0.0548 (ns)<br>0.0267 (*)<br>0.0074 (**)<br>0.0022 (**)<br>0.0090 (**)<br>0.0255 (*)<br>0.0123 (*)<br>0.0296 (*)<br>0.0048 (**)<br>0.0046 (**)
Sex	F(1,10) = 46.75	<0.0001 (***)		
Interaction	F(25,250) = 3.533	<0.0001 (***)		
Subject	F(10,250) = 33.43	<0.0001 (***)		
**Change in Body Weight (%)**				
**Females**				
Exercise	F(1,10) = 0.02512	0.8772 (ns)	Day 7: Controls: *vs.* Runners<br>Day 14: Controls: *vs.* Runners<br>Day 21: Controls: *vs.* Runners<br>Day 28: Controls: *vs.* Runners	0.9972 (ns)<br>>0.9999 (ns)<br>0.9993 (ns)<br>0.9578 (ns)
Time	F(2.066,20.66) = 0.7143	0.5056 (ns)		
Interaction	F(3,30) = 0.7811	0.5138 (ns)		
**Males**				
Exercise	F(1,10) = 2.532	0.1426 (ns)	Day 7: Controls: *vs.* Runners<br>Day 14: Controls: *vs.* Runners<br>Day 21: Controls: *vs.* Runners<br>Day 28: Controls: *vs.* Runners	0.9908 (ns)<br>0.9203 (ns)<br>0.9495 (ns)<br>0.2720 (ns)
Time	F(2.045,17.72) = 2.841	0.0842 (ns)		
Interaction	F(3,26) = 2.389	0.0917 (ns)		

Food Consumption (g)				
**Females**				
Exercise	F(1,10) = 38.89	<0.0001 (***)	Day 7: Controls: *vs.* Runners<br>Day 14: Controls: *vs.* Runners<br>Day 21: Controls: *vs.* Runners<br>Day 28: Controls: *vs.* Runners	0.2264 (ns)<br>0.0046 (**)<br>0.0081 (**)<br>0.0122 (*)
Time	F(1.994,18.61) =10.24	0.0010 (**)		
Interaction	F(3,28) = 4.581	0.0099 (**)		
**Males**				
Exercise	F(1,10) = 2.179	0.1707 (ns)	Day 7: Controls: *vs.* Runners<br>Day 14: Controls: *vs.* Runners<br>Day 21: Controls: *vs.* Runners<br>Day 28: Controls: *vs.* Runners	>0.9999 (ns)<br>0.1263 (ns)<br>0.0394 (*)<br>>0.9999 (ns)
Time	F(1.765,17.65) = 2.569	0.1099 (ns)		
Interaction	F(3,30) = 2.630	0.0683 (ns)		

Supplemental Table 1. Summary of 2-way ANOVA results for daily and total wheel running activity, change in body weight, and food consumption, assessing the effects of time, exercise and sex on these parameters in mice (*$p < 0.05$, **$p < 0.01$, ***$p < 0.001$, n = 6 mice per group). Significant effects were seen for time and sex in daily and total running activity, as well as food consumption in females. Sidák's *post hoc* comparisons of relevant selected pairs confirmed differences between males and females in daily running activity in the dark phase and total running activity, as well as greater food consumption in female runners *vs*. controls.

Supplemental Table 2: Statistical Analysis for Motor Performance Tests				
	2-way ANOVA (full-model)		*Post hoc* test	
	**F(DFn,DFd)**	***p* value**	**Sidák's selected comparisons**	***p* value**
**Open Field**				
**Distance (m)**				
Exercise	F(1,20) = 5.826	0.0255 (*)	Controls:Female *vs.* Runners:Female<br>Controls:Male *vs.* Runners:Male<br>Controls:Female *vs.* Controls:Male<br>Runners:Female *vs.* Runners:Male	0.8733 (ns)<br>0.0726 (ns)<br>0.9895 (ns)<br>0.1727 (ns)
Sex	F(1,20) = 3.233	0.0873 (ns)		
Interaction	F(1,20) = 1.456	0.2417 (ns)		
**Velocity (cm/s)**				
Exercise	F(1,22) = 5.191	0.0328 (*)	10 min: Controls *vs.* Runners<br>20 min Controls *vs.* Runners<br>30 min: Controls *vs.* Runners<br>40 min Controls *vs.* Runners<br>50 min: Controls *vs.* Runners<br>60 min Controls *vs.* Runners	0.4065 (ns)<br>0.1995 (ns)<br>0.0569 (ns)<br>0.6797 (ns)<br>0.4689 (ns)<br>0.8349 (ns)
Time	F(3.047,67.030) = 51.18	<0.001 (***)		
Interaction	F(5,110) = 0.929	0.4650 (ns)		
Subject	F(22,110) = 9.815	<0.001 (***)		
**Time moving (s)**				
Exercise	F(1,20) = 4.165	0.0547 (ns)	Controls:Female *vs.* Runners:Female<br>Controls:Male *vs.* Runners:Male<br>Controls:Female *vs.* Controls:Male<br>Runners:Female *vs.* Runners:Male	0.8941 (ns)<br>0.2356 (ns)<br>0.5161 (ns)<br>0.0898 (ns)
Sex	F(1,20) = 7.586	0.0122 (*)		
Interaction	F(1,20) = 0.5198	0.4793 (ns)		
**Center preference (%)**				
Exercise	F(1,20) = 0.1863	0.6707 (ns)	Controls:Female *vs.* Runners:Female	

Sex	F(1,20) = 3.260	0.0861 (ns)	Controls:Male *vs.* Runners:Male<br>Controls:Female *vs.* Controls:Male<br>Runners:Female *vs.* Runners:Male	0.9698 (ns)<br>>0.9999 (ns)<br>0.7837 (ns)<br>0.4577 (ns)
Interaction	F(1,20) = 0.1277	0.7246 (ns)		
**Vertical Pole**				
**Time to descend (s)**				
Exercise	F(1,20) = 11.620	0.0028 (**)	Controls:Female *vs.* Runners:Female<br>Controls:Male *vs.* Runners:Male<br>Controls:Female *vs.* Controls:Male<br>Runners:Female *vs.* Runners:Male	0.0170 (*)<br>0.4158 (ns)<br>0.8442 (ns)<br>0.9297 (ns)
Sex	F(1,20) = 0.0205	0.8875 (ns)		
Interaction	F(1,20) = 1.320	0.2641 (ns)		
**Adhesive Tape Removal**				
**Time to first approach (s)**				
Exercise	F(1,20) = 0.4855	0.4940 (ns)	Controls:Female *vs.* Runners:Female<br>Controls:Male *vs.* Runners:Male<br>Controls:Female *vs.* Controls:Male<br>Runners:Female *vs.* Runners:Male	0.9985 (ns)<br>0.9230 (ns)<br>0.0408 (*)<br>0.1111 (ns)
Sex	F(1,20) = 13.420	0.0015 (**)		
Interaction	F(1,20) = 0.1137	0.7395 (ns)		
**First sticker removal (s)**				
Exercise	F(1,20) = 3.242	0.0869 (ns)	Controls:Female *vs.* Runners:Female<br>Controls:Male *vs.* Runners:Male<br>Controls:Female *vs.* Controls:Male<br>Runners:Female *vs.* Runners:Male	0.3028 (ns)<br>0.9192 (ns)<br>0.7869 (ns)<br>0.1871 (ns)
Sex	F(1,20) = 4.804	0.0404 (*)		
Interaction	F(1,20) = 0.5647	0.4611 (ns)		
**Second sticker removal (s)**				
Exercise	F(1,20) = 3.423	0.0791 (ns)	Controls:Female *vs.* Runners:Female<br>Controls:Male *vs.* Runners:Male<br>Controls:Female *vs.* Controls:Male<br>Runners:Female *vs.* Runners:Male	0.0784 (ns)<br>>0.9999 (ns)<br>>0.9999 (ns)<br>0.0796 (ns)
Sex	F(1,20) = 3.385	0.0807 (ns)		
Interaction	F(1,20) = 2.951	0.1013 (ns)		
**Grip Strength**				
**Forelimbs Force/BW (g/g)**				
Exercise	F(1,20) = 0.0770	0.7843 (ns)	Controls:Female *vs.* Runners:Female<br>Controls:Male *vs.* Runners:Male<br>Controls:Female *vs.* Controls:Male<br>Runners:Female *vs.* Runners:Male	0.9458 (ns)<br>0.9981 (ns)<br>0.9547 (ns)<br>0.4441 (ns
Sex	F(1,20) = 2.370	0.1394 (ns)		
Interaction	F(1,20) = 0.4284	0.5202 (ns)		
**All limbs Force/BW (g/g)**				
Exercise	F(1,20) = 0.6079	0.4447 (ns)	Controls:Female *vs.* Runners:Female<br>Controls:Male *vs.* Runners:Male<br>Controls:Female *vs.* Controls:Male<br>Runners:Female *vs.* Runners:Male	0.9141 (ns)<br>0.9949 (ns)<br>0.1952 (ns)<br>0.3858 (ns)
Sex	F(1,20) = 6.873	0.0163 (*)		
Interaction	F(1,20) = 0.0836	0.7754 (ns)		

Supplemental Table 2. Summary of 2-way ANOVA results for motor and sensorimotor behavioral tests, assessing the effects of exercise and sex on performance parameters in mice. Tests include locomotor activity in an open field, vertical pole test, adhesive tape removal test, and grip strength test (*$p < 0.05$, **$p < 0.01$, ***$p < 0.001$, n = 6 mice per group). Significant effects in 2-way ANOVA are seen in the influence of exercise on motor performance (e.g., distance, velocity, and vertical pole descent), and for sex in aspects of some parameters (time moving in the open field, tape removal, and grip strength). Sidák's *post hoc* comparisons of relevant selected pairs, however, reveal no differences between males and females except for the time to first approach in the adhesive tape removal test, enabling data from males and females to be combined for all other analyses.
